# Mechanics of Biological Tissues and Biomaterials: Current Trends

**DOI:** 10.3390/ma8074505

**Published:** 2015-07-21

**Authors:** Amir A. Zadpoor

**Affiliations:** Department of Biomechanical Engineering, Faculty of Mechanical, Maritime, and Materials Engineering, Delft University of Technology (TU Delft), Mekelweg 2, Delft 2628CD, The Netherlands; E-Mail: a.a.zadpoor@tudelft.nl; Tel.: +31-15-278-1021; Fax: +31-15-278-4717

**Keywords:** mechanical behavior, biological tissues, biomaterials, measurement techniques, constitutive modeling

## Abstract

Investigation of the mechanical behavior of biological tissues and biomaterials has been an active area of research for several decades. However, in recent years, the enthusiasm in understanding the mechanical behavior of biological tissues and biomaterials has increased significantly due to the development of novel biomaterials for new fields of application, along with the emergence of advanced computational techniques. The current Special Issue is a collection of studies that address various topics within the general theme of “mechanics of biomaterials”. This editorial aims to present the context within which the studies of this Special Issue could be better understood. I, therefore, try to identify some of the most important research trends in the study of the mechanical behavior of biological tissues and biomaterials.

## 1. Introduction

The mechanical behavior of biological tissues and biomaterials has been intensively studied for decades, but has recently been receiving increasing attention. The mechanical properties of biological tissues were traditionally studied within the biomechanics community. However, the biomaterials community is becoming interested in this field of research through analyzing the most important predictors of biomaterials suitability, especially their stiffness and strength. During the last decade, our ability to characterize and analyze biological tissues, on the one hand, and design and synthesize “multi-functional biomaterials”, on the other hand, has improved substantially. In many cases, these multi-functional biomaterials either replace or enable the regeneration of damaged tissues. There are, therefore, either temporary or permanent interactions between (evolving) tissues and the multi-functional biomaterials that come in contact with them. These interactions take several forms but mechanical interaction is one of the most important types, particularly for load-bearing tissues, such as musculoskeletal tissues. Within the context of these developments, a wider range of researchers have become interested in studying the mechanical interactions between tissues and biomaterials. In many cases, this means the study of the mechanical behavior of both biological tissues and biomaterials, not only to determine the basic mechanical properties, but also to extract the type of data that is needed for advanced constitutive modeling of those materials. Moreover, the multi-functional nature of many biomaterials conveys that their mechanical properties are not only important from the mechanical and load-bearing viewpoints, but also in the way that they influence their other bio-functionalities. There are indeed many examples where the mechanical properties of biomaterials influence and/or regulate their biological performance. For example, it is shown that the physical and mechanical properties of the matrix on which stem cells are cultured could influence the behavior of stem cells [1,2]. Moreover, post-manufacturing treatments of biomaterials, which are usually aimed at improving one or more functionalities of the biomaterials, could influence the mechanical function of biomaterials as well. That is why the different functionalities of biomaterials need to be simultaneously studied. Finally, there is a recent trend in the “rational design” of biomaterials, where materials with specific micro-architectures are designed to achieve specific mechanical and biological properties. Given the recent advances in 3D printing and additive manufacturing, it is now possible to manufacture almost any such design, meaning that an unlimited number of rationally designed biomaterials have become available that need to be studied, among other aspects, from the mechanical viewpoint.

Given all the above-mentioned developments, we felt it is a good time to dedicate a Special Issue of the journal *Materials* to the study of the mechanical behavior of biological tissues and biomaterials. Many authors from all around the world contributed their latest research, which were subsequently reviewed to select the studies that form this Special Issue. This editorial tries to present the context within which these selected studies could be better understood.

## 2. Mechanics of Biological Tissues

There are several reasons why one may be interested in the mechanical behavior of biological tissues. The relevance of such studies is very clear for skeletal tissues, such as bone, cartilage, and tendon, whose main functions are structural. That is why many of the earliest studies on the mechanical behavior of biological tissues were focused on skeletal tissues. To date, skeletal tissues are among the most intensively studied biological tissues in terms of their mechanical behavior. However, many more types of tissues are now being studied, including brain [3,4,5], liver [6,7], muscle [8,9], and adipose tissue [10]. In the remainder of this section, I will highlight three of the most important areas where the mechanical properties of tissues are needed.

### 2.1. Constitutive Modeling of Biological Tissues

Advanced materials models are needed when describing the mechanical response of biological tissues to multi-axial loading. That is partly due to the heterogeneity of the mechanical properties, anisotropy, time-dependency of the mechanical behavior, presence of several phases (fluid, solid, ions, *etc.*), and adaptation of mechanical properties to mechanical loading. That is particularly important when developing computational models of tissues. Since most biological tissues are strongly hierarchical, it is particularly interesting to relate the microstructure of tissues to their macro-scale mechanical behavior. In the present issue, Taghizadeh *et al.* [11] relate the mechanical behavior of aortic tissue to the lamellar structure. In another study, Li *et al.* [12] have developed a highly stretchable substrate made from fugitive glue that could be used to study “the effects of large strains on biological samples”.

### 2.2. Tissue Regeneration

Regeneration of damaged tissues using tissue engineering and regenerative medicine approaches is an important aim pursued by the biomedical engineering community. In order to regenerate tissues, one needs to provide the proper environment for tissue regeneration including media (*i.e.*, scaffolds, gels) that are mechanically strong enough to support the process of tissue regeneration. At the same time, tissue engineering scaffolds should not be overly stiff, because they might otherwise impede the regeneration of tissues [13,14]. It is therefore natural to ask: “What would are the optimal range of the mechanical properties of tissue engineering scaffolds and gels?” One approach is to characterize the native tissue to gain some insight into the expected range of mechanical properties [13,14]. This approach has some limitations, because the mechanical properties required to optimally support the process of tissue regeneration may not necessarily be the same as those of the native tissue. Despite those limitations, the properties of the native tissue are, in many cases, the best available starting point. Moreover, the mechanical properties of biological tissues could be used for diagnosis of diseases that manifest themselves in terms of changes in the mechanical properties of tissues. In this issue, Nesbitt *et al.* [15] study the mechanical behavior of the skin tissue and how collagen fibrils respond to mechanical loading. This type of information could be potentially useful both for diagnosis of skin diseases and for tissue engineering applications.

### 2.3. Tissue Damage and Trauma

Mechanical loading of tissues combined with underlying diseases could lead to tissue damage. This includes not only the non-physiological loading that is experienced in traumatic events but also physiological loading of tissues when a chronic disease such as osteoporosis is present. In a chronic disease such as osteoporosis, one is interested in knowing what level of mechanical loading could the bones tolerate without risking osteoporotic fracture. Knowing the answer to that question requires information regarding the mechanical properties of bones. Similarly, studying the changes in the mechanical properties of cartilage is crucial when studying osteoarthritis. Indeed, it has been demonstrated that changes in the mechanical properties of cartilage are one of the first indicators of osteoarthritis onset [16]. As for trauma, one is concerned about how biological tissues respond to non-physiological loading. In this issue, Weed *et al.* [17] report on the mechanical isotropy of porcine lung parenchyma, which is an important property when deciding what kind of constitutive modeling approach should be used for analysis of the mechanical behavior of that tissue. The mechanical behavior of the lung tissue is important, for example, when studying the pulmonary injuries caused by trauma.

## 3. Mechanics of Biomaterials

### 3.1. Implants

Implants that are aimed to stay in the human body for a long time were among the first biomaterials. It is important to ensure that the implants do not fail under their service load. Therefore, the mechanical properties of implants, such as static mechanical properties and fatigue behavior, need to be studied. In addition to the implants themselves, the biomaterials that are used for fixation of the implants or filling the cavities inside (hard) tissue need to satisfy certain requirements in terms of their mechanical properties. Finally, the mechanical properties of implants could have consequences for their function even when there is no risk of mechanical failure. Perhaps the most important example is the stress shielding phenomenon [18], where overly stiff implants could cause tissue resorption, implant loosening, and ultimately implant failure. All these concerns have motivated the study of the mechanical behavior of implant systems. In the current issue, Maurer *et al.* [19] study the mechanical behavior of different designs of prosthetic meshes that are used to repair hernia and pelvic organ prolapse. In another study, Weiss and Mitevski [20] report on the microstructure and deformation of different designs of CoCr coronary stents. Bone cements are the subjects of two other studies published in the current Special Issue. In the first study, Geffers *et al.* [21] review the strategies for reinforcement of calcium phosphate cements. In the second study, Jiang *et al.* [22] investigate the effects of adding mineralized collagen on the mechanical properties and cytocompatibility of PMMA (polymethyl methacrylate) bone cements.

### 3.2. Biomaterials for Tissue Regeneration

As discussed in Section 2.2, the biomaterials that are used to facilitate tissue regeneration need to satisfy a set of requirements concerning their physical, mechanical, and biological properties. Assuming we know the expected range of the mechanical properties of the tissues that need to be regenerated, the next step is to develop biomaterials that exhibit the desired mechanical properties while satisfying other requirements. Characterizing the mechanical properties of tissue engineering scaffolds is therefore an important line of research, as is clear from the large number of related studies appearing in the current issue. Goncalves *et al.* [23] study hybrid membranes of PLLA (poly-l-lactide) and collagen and how their production techniques influence the mechanical properties and osteoinduction ability of the resulting bone tissue engineering scaffolds. In another study, Wang *et al.* [24] used phase separation to fabricate bone tissue engineering scaffolds based on poly (lactide-*co*-glycolide) and tight-coated with gelatin. The effects of gelatin modification on hydrophilicity and mechanical properties of the scaffolds were investigated. Teng *et al.* [25] developed porous films whose wettability and adhesion could be tuned. This technology has potential applications in tissue engineering of various types of tissues. Finally, Chan *et al.* [26] combined polypropylene with boron nitride and nanohydroxyapatite to develop biocomposites aimed for application as bone substitutes. The effects of above-mentioned reinforcements on the mechanical properties and biocompatibility of the resulting biomaterials were studied.

### 3.3. Biofabrication

The application of advanced manufacturing techniques, such as 3D printing and additive manufacturing, in the fabrication of medical devices and biomaterials is often called biofabrication. Biofabrication techniques have enabled us to manufacture new categories of biomaterials that intimately interact with cells and organs and could have arbitrarily complex micro-architectures. There is a direct relationship between the micro-architecture of biomaterials and their physical, biological, and mechanical properties [27]. The micro-architecture of such biomaterials could therefore be used to create unique combinations of biological, mechanical, and physical properties. Many research groups worldwide are researching the mechanical properties of additively manufactured biomaterials. For example, in the current issue, Ahmadi *et al.* [28] study the relationship between the type of repeating unit cell and the static and morphological properties of selective laser melted porous biomaterials.

### 3.4. Soft Biomaterials

Soft biomaterials and matrices particularly specific types of (hydro-) gels have been in the center of recent attention of many research groups. Among other applications, these soft biomaterials could provide suitable environments for tissue regeneration. However, the mechanical properties of many types of hydrogels are not high enough to enable them provide enough mechanical support for tissue regeneration. That is why improving the mechanical properties of gels is particularly important and new variants of (hydro-) gels with significantly improved mechanical properties have been developed during the last few years, see, for example, [29]. In addition to improving the mechanical properties of gels, there are other mechanical aspects that need to be fully understood. One example is the swelling behavior of gels in presence of water and ions and development of computational models and constitutive equations that could simulate the swelling behavior.

A number of studies appearing in the current special issue study the various aspects of the mechanical, physical, and biological behavior of soft biomaterials including (hydro-) gels. Baniasadi and Minary-Jolandan [30] report on development and mechanical characterization of a composite hydrogel based on alginate and collagen. Lin and Gu [31] study the effects of crosslink density and stiffness on the mechanical properties of type I collagen gel. In another study, Moreno-Arotzena *et al.* [25] study the properties of collagen and fibrin gels aimed for application in wound healing.

## 4. Conclusions

Study of the mechanical behavior of biological tissues and biomaterials has been flourishing during the last few years partly due to recent developments in the biomechanics and biomaterials communities. Increasingly, the study of the mechanical behavior is combined with the study of the other aspects of biomaterial functionality. The studies appearing in the current Special Issue contribute towards better understanding of the various aspects of the mechanical behavior of both biological tissues and biomaterials.
